# *Trypanosoma cruzi* infection status in birthing individuals in three endemic provinces of Argentina: a descriptive cross-sectional study

**DOI:** 10.1590/0037-8682-0328-2025

**Published:** 2026-03-02

**Authors:** María Ayelen Toscani, Emmaria Danesi, Karen Klein, Luz Gibbons, Candela Belén Stella, Pablo Elías Gulayin, Mabel Berrueta, Eric Dumonteil, Claudia Herrera, Pierre Buekens, María Luisa Cafferata, BETTY Collaborative Group

**Affiliations:** 1 Instituto de Efectividad Clínica y Sanitaria (IECS), Ciudad Autónoma de Buenos Aires, Argentina.; 2 Instituto Nacional de Parasitología "Dr. Mario Fatala Chabén" (INP), Ciudad Autónoma de Buenos Aires, Argentina.; 3 Tulane University, Celia Scott Weatherhead School of Public Health and Tropical Medicine, New Orleans, USA.

**Keywords:** *Trypanosoma cruzi* infection, Chagas disease, Diagnostic serology, Postpartum

## Abstract

**Background:**

Chagas disease remains a public health challenge with underexplored prevalence and diagnosis in endemic areas. We assessed *Trypanosoma cruzi* infection screening among birthing individuals in three endemic provinces in northern Argentina.

**Methods:**

*T. cruzi*-seropositive birthing individuals with at least one positive or indeterminate test were enrolled during the postpartum period to confirm seropositivity by indirect hemagglutination (IHA) and enzyme-linked immunosorbent (ELISA) assays.

**Results:**

Among 40,035 deliveries, the *T. cruzi* infection rate was 1.9%. Confirmatory serology revealed 77.6% positivity, 9.3% negativity, and 13.0% discordance.

**Conclusions:**

These findings highlight the need for confirmatory testing and improved diagnostic strategies in endemic regions.

Chagas disease (American trypanosomiasis), caused by the protozoan parasite *Trypanosoma cruzi*, remains one of the most neglected tropical diseases despite being discovered over a century ago.

Despite the efforts made to control Chagas disease as a public health problem in Latin America, over seven million individuals are infected, including approximately 1,764,000 women of reproductive age^1^. In Argentina, the estimated prevalence of Chagas disease among pregnant women ranges from 1.4 to 1.8%. Currently, vertical transmission represents the primary route of new infections in Argentina and several countries in the region^1^, with an average transmission rate of approximately 5%-8%^2^.

The Latin American and Argentinian Chagas guidelines recommend screening pregnant women during antenatal care, ideally at the first prenatal visit, and confirming chronic *T. cruzi* infection with at least two positive serological tests, with the addition of a third test when results are discordant. When antenatal screening is unavailable, testing is recommended at delivery or before discharge from the healthcare facility^3^ ^,^ ^4^. Official data in Argentina show that diagnostic coverage varies across different provinces^2^.

As part of the BETTY trial (a double-blind, non-inferiority randomized clinical trial)^5^, this cross-sectional descriptive study aimed to estimate *T. cruzi* infection status and describe the sociodemographic profiles of birthing individuals in three endemic provinces in northern Argentina.

The study was conducted at four public healthcare facilities: Hospital J.C. Perrando (Resistencia, Chaco Province), Centro Integral de Salud La Banda (La Banda, Santiago del Estero Province), Hospital Regional Dr. Ramón Carrillo (Santiago del Estero capital, Santiago del Estero Province), and the Instituto de Maternidad y Ginecología Nuestra Señora de las Mercedes (San Miguel de Tucumán, Tucumán Province). Each facility has a reference laboratory specialized in Chagas disease.

Birthing individuals with a history of at least one positive or indeterminate *T. cruzi* serological test who delivered at the study sites between June 2019 and August 2022 were identified as potential participants and invited to participate. After providing written informed consent, participants were screened for eligibility. After enrollment, blood samples were collected to assess *T. cruzi* seropositivity using two serological tests: the indirect hemagglutination (IHA) assay and an enzyme-linked immunosorbent assay (ELISA). Anti-*T. cruzi* antibodies (IgG) were detected using two Wiener Laboratory kits (Rosario, Argentina): Chagastest IHA and Chagastest ELISA Recombinant v3.0. The BETTY trial followed standard operating procedures based on the manufacturer’s instructions to ensure consistency in all laboratories and the quality of the results. Participants with reactive results in both tests were considered seropositive, and those with one reactive result and either a nonreactive or indeterminate result were defined as discordant. Participants with nonreactive results in both tests were considered non-infected. According to the BETTY trial protocol, no third confirmatory test was performed in cases of discordant results^5^.

Data on *T. cruzi* prenatal screening, previous serological test results, age, nationality, education level, and place of residence (urban/rural) were obtained from clinical records, antenatal cards, and participant interviews. The number of deliveries was recorded at each study site.

The number of *T. cruzi*-seropositive birthing individuals at delivery, the subset without a previous diagnosis, confirmatory serological test results, and participant characteristics were reported globally and by province. Frequencies and proportions were reported for all variables. R version 4.0.3 was used for data analysis.

The BETTY trial was approved by the Tulane University Institutional Review Board (USA) and by the Comité de Ética en la Investigación, Hospital Dr. Julio C. Perrando (Chaco Province); the Comité Institucional de Ética en Investigación en Salud, Ministerio de Salud (Santiago del Estero Province); and the Comité de Ética de Investigación en Salud del Sistema Provincial de Salud, Ministerio de Salud Pública (Tucumán Province).

Among the 40,035 reported deliveries during the study period, 2,426 (6.1%) had no documented diagnosis of *T. cruzi* infection at delivery, and 748 (1.9%) had at least one positive or indeterminate serological result (Figure 1 and Table 1). Of these 748 birthing individuals, 419 (56.0%) provided informed consent and were screened for eligibility according to the BETTY trial criteria^5^, resulting in the enrollment of 255 participants (60.9%). Confirmatory serology was performed in 246 of the 255 enrolled participants. Among these, 191 (77.6%) were confirmed as seropositive by both IHA and ELISA. *T. cruzi* infection was not confirmed in the remaining 55 (22.4%) participants, including 23 (9.3%) with negative results and 32 (13.0%) with discordant results. Most discordant cases (84.4%) were from Santiago del Estero, where the majority showed negative ELISA results (Table 1).

**FIGURE 1:**
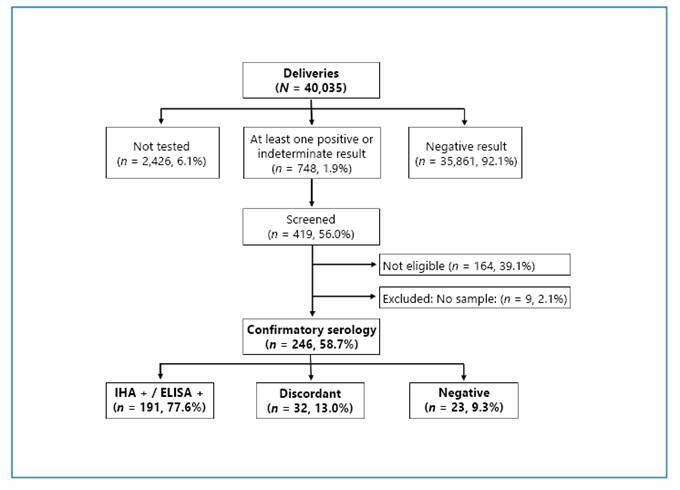
Flowchart of serological screening and confirmatory diagnosis. Main exclusion reasons included residence outside the follow-up area, previous trypanocidal treatment, and female sterilization.

**TABLE 1:** *T. cruzi* infection rate at delivery and confirmatory serology among participants enrolled in the BETTY trial.

	Chaco		Santiago del Estero		Tucumán		Total	
	*n/N*	%	*n/N*	%	*n/N*	%	*n/N*	%
Screening at delivery*								
With at least one positive or indeterminate serological test	130/6,213	2.1	523/14,411	3.6	95/19,411	0.5	748/40,035	1.9
Without a previous serological test	337/6,213	5.4	966/14,411	6.7	1,123/19,411	5.8	2,426/40,035	6.1
**Confirmatory serology** ^#^								
Both tests +	43/46	93.5	110/154	71.4	38/46	82.6	191/246	77.6
Both tests -	1/46	2.2	17/154	11.0	5/46	10.9	23/246	9.3
**Discordant**								
IHA -/ELISA +	0/46	0.0	5/154	3.2	2/46	4.3	7/246	2.8
IHA +/ELISA -	1/46	2.2	14/154	9.1	1/46	2.2	16/246	6.5
At least one indeterminate	1/46	2.2	8/154	5.2	0/46	0.0	9/246	3.7

The two sections of this table use different denominators, which are not directly comparable. * Screening at delivery: ***N:*** corresponds to the total number of reported deliveries by province and the overall number during the study period. # Confirmatory serology: ***N:*** corresponds to the total number of participants who provided a blood sample for confirmatory testing, analyzed by province and overall. Blood samples were collected from 246 of the 255 participants enrolled in the BETTY trial.

Forty-five percent of the participants were aged between 26 and 34 years, 98.4% were Argentinians, and 58.0% lived in urban areas. Over 80% of the participants from Tucumán and Chaco lived in urban dwellings, whereas 58.5% lived in rural dwellings in Santiago del Estero. Regarding education, 84.7% of the participants had completed primary school (Table 2).

**TABLE 2:** Characteristics of participants.

	Chaco		Santiago del Estero		Tucumán		Total	
	**(*N* = 49)**		**(*N* = 159)**		**(*N* = 47)**		**(*N* = 255)**	
	*n/N*	%	*n/N*	%	*n/N*	%	*n/N*	%
**Age (years)**								
16-18	1/49	2.0	16/159	10.1	4/47	8.5	21/255	8.2
19-25	15/49	30.6	36/159	22.6	12/47	25.5	63/255	24.7
26-34	24/49	49.0	70/159	44.0	21/47	44.7	115/255	45.1
≥35	9/49	18.4	37/159	23.3	10/47	21.3	56/255	22.0
**Nationality**								
Argentina	48/49	98.0	159/159	100.0	44/47	93.6	251/255	98.4
Bolivian	1/49	2.0	0/159	0.0	3/47	6.4	4/255	1.6
Place of residence*								
Urban	43/49	87.8	66/159	41.5	39/47	83.0	148/255	58.0
Rural	6/49	12.2	93/159	58.5	8/47	17.0	107/255	42.0
**Education**								
Informal education	0/49	0.0	2/159	1.3	0/47	0.0	2/255	0.8
Incomplete primary school	6/49	12.2	31/159	19.5	0/47	0.0	37/255	14.5
Complete primary school	10/49	20.4	62/159	39.0	23/47	48.9	95/255	37.3
Incomplete secondary school	20/49	40.8	40/159	25.2	5/47	10.6	65/255	25.5
Complete secondary school	10/49	20.4	19/159	11.9	17/47	36.2	46/255	18.0
Tertiary school or higher	3/49	6.1	5/159	3.1	2/47	4.3	10/255	3.9

Participant sociodemographic characteristics, analyzed by province and overall. *Areas with 2,000 or more inhabitants were classified as urban areas.

This study provides baseline data that offer a unique opportunity to assess the current epidemiology of *T. cruzi* infection during the postpartum period in three endemic provinces of Argentina.

Given that these four healthcare facilities serve as provincial reference centers, providing care to birthing individuals from across each province, the observed seroprevalence rates may reasonably be considered representative of the populations of birthing individuals in each jurisdiction. The positivity rates observed in Tucumán and Santiago del Estero were closely aligned with the official national estimates reported in 2019 (0.6% and 2.6%, respectively). In contrast, Chaco showed a significant deviation from historically reported rates, particularly from the provincial estimate of 11.1% in 2019^6^, suggesting a potentially higher prevalence in areas not included in the BETTY trial.

In Argentina, routine screening for anti*-T. cruzi* antibodies during pregnancy is recommended to identify maternal infection and ensure early detection and follow-up of newborns at risk of vertical transmission. A confirmed diagnosis requires at least two positive serological tests, and the infection status must be systematically documented in clinical records, antenatal cards, and the national epidemiological surveillance system^3^ ^,^ ^4^ ^,^ ^7^ ^,^ ^8^. However, official data reveal significant gaps in implementation, with 12-37% of pregnant individuals remaining undiagnosed^2^, which is higher than the proportion observed in this study. This difference may be attributed to potential underreporting by the National Health Information System, highlighting the need for enhanced adherence to national recommendations.

Among the participants who underwent confirmatory serology, 9.3% tested negative and 13.0% exhibited discordant results. This finding is particularly noteworthy because all participants had at least one positive serology as a previous antecedent. As clinical records and antenatal cards document serological results as either positive or negative, specific types and semi-quantitative results from the tests are not always available. Negative results could be attributed to previous screening using a single serological test or a positive IHA result with lower specificity, highlighting the need for more accurate diagnostic tests. Our definition of discordance is conservative, relying solely on the lack of agreement between confirmatory tests conducted under strict quality controls. The observed prevalence of discordant serologies is a significant concern, with most cases concentrated in Santiago del Estero. Although no previous studies were found to support this finding, experienced professionals have mentioned frequent discordant results among individuals from this province (personal communication). In addition, studies from other regions have reported high rates of discordant serology; however, in these cases, discordance was defined based on three to five immunoassays^9^ ^,^ ^10^. A study conducted at a blood bank in Chaco, near the border of Santiago del Estero, reported a 6.5% rate of inconclusive *T. cruzi* infection diagnoses^10^, which was lower than that observed among our participants from Chaco and significantly lower than that observed in participants from Santiago del Estero. Discordant serology may be attributed to varying reactivity to antigens present in commercial assays and exposure to different discrete typing units (DTUs) and strains across several regions^11^. Another hypothesis suggests that discordant serologies may be linked to the progression of infection within an individual. Two studies have demonstrated that immunological responses indicative of resolved infection are associated with low antibody titers that decline toward seronegativization^9^ ^,^ ^12^. This process is accompanied by persistently negative polymerase chain reaction (PCR) results, which may indicate parasite elimination. Previous studies have shown lower and declining rates of parasite detection by PCR in individuals with low seroreactivity and decreasing antibody titers over time^12^. In addition, in certain areas of northern Argentina, Chagas disease coexists with leishmaniasis, caused by trypanosomatids of the genus *Leishmania*, which may lead to serological cross-reactivity with several *T. cruzi* antigens^13^. Our discordant results highlight the importance of conducting a third confirmatory test in this population, particularly in Santiago del Estero. Ensuring a confirmatory diagnosis of *T. cruzi* infection in childbearing individuals remains a crucial public health priority in Argentina.

In line with previous research, our findings showed that more than half of the participants lived in urban areas, highlighting that *T. cruzi* infection is not confined to rural or vector-endemic settings but is also widespread in urban regions^14^ ^,^ ^15^. Although data on previous places of residence were not collected, some participants may have acquired the infection in rural areas, reflecting the effects of progressive urbanization and migration, which have contributed to the emergence of Chagas disease as a significant urban health problem worldwide^16^.

This study addresses a significant gap in the literature, given the limited data on *T. cruzi* infection among childbearing individuals during childbirth in Argentina^11^. Its main strengths include rigorous data collection, monitoring, and standardized laboratory procedures. However, this study has several limitations. Participants with confirmed *T. cruzi* infections may not fully represent the broader seropositive population giving birth in these regions, as all four participating healthcare facilities belong to the public health subsystem. Moreover, the inclusion and exclusion criteria may limit the generalizability of the findings to a wider provincial population. Finally, the study could not confirm *T. cruzi* infection status in cases with discordant results because a third serological test was not performed.

Chagas disease remains a neglected public health problem in Latin America. This study provides valuable data on the prenatal screening and serological profiles of childbearing individuals in endemic regions of Argentina. Understanding the serological and socioeconomic characteristics of this population is essential for designing effective preventive programs and interventions. These efforts can improve the diagnostic and treatment strategies for both childbearing individuals and their offspring, thereby advancing primary and secondary prevention goals. Furthermore, this knowledge may guide new research directions and offer valuable insights for public health decision makers to reduce the burden of Chagas disease.

## TRIAL REGISTRATION

ClinicalTrials.gov. Identifier: NCT03672487. Registered on 14 September 2018.

## DECLARATION OF THE USE OF GENERATIVE AI AND AI-ASSISTED TECHNOLOGIES IN THE WRITING PROCESS

The authors used Grammarly (Grammarly Inc., USA) and ChatGPT (OpenAI, San Francisco, CA, USA) solely for English language editing and stylistic improvement. These tools did not generate or modify the scientific content, and all interpretations, analyses, and conclusions remain the sole responsibility of the authors. 
